# Brown fat-associated postprandial thermogenesis in humans: Different effects of isocaloric meals rich in carbohydrate, fat, and protein

**DOI:** 10.3389/fnut.2022.1040444

**Published:** 2022-10-26

**Authors:** Sayuri Aita, Mami Matsushita, Takeshi Yoneshiro, Takuya Hatano, Toshimitsu Kameya, Iwao Ohkubo, Masayuki Saito

**Affiliations:** ^1^Department of Nutrition, School of Nursing and Nutrition, Tenshi Collage, Sapporo, Japan; ^2^Department of Food and Health Sciences, College of Life Sciences, Ibaraki Christian University, Hitachi, Japan; ^3^Division of Metabolic Medicine, Research Center for Advanced Science and Technology, The University of Tokyo, Tokyo, Japan; ^4^Kushiro Health Care Center, Hokkaido Government, Kushiro, Japan; ^5^LSI Sapporo Clinic, Sapporo, Japan; ^6^Laboratory of Biochemistry, Faculty of Veterinary Medicine, Hokkaido University, Sapporo, Japan

**Keywords:** brown adipose tissue, carbohydrate-rich meal, diet-induced thermogenesis, fat-rich meal, humans, protein-rich meal

## Abstract

The increase of whole-body energy expenditure seen after a single meal ingestion, referred to as diet-induced thermogenesis (DIT), substantially varies depending on the meal’s macronutrient composition. Brown adipose tissue (BAT), a site of non-shivering thermogenesis, was reported to be involved in DIT. To examine the effects of meal composition on BAT-associated DIT in humans, healthy male participants underwent fluorodeoxyglucose–positron emission tomography to assess BAT activity, and respiratory gas analysis for 2 h after ingestion of a carbohydrate-, protein-, or fat-rich meal (C-meal, P-meal, and F-meal, respectively). The calculated DIT at 2 h was 6.44 ± 2.01%, 3.49 ± 2.00%, and 2.32 ± 0.90% of the ingested energy after the P-meal, C-meal, and F-meal, respectively. The DIT after C-meal ingestion correlated positively with BAT activity (*P* = 0.011), and was approximately twice greater in the group with high-BAT activity than in the group with low-BAT activity (4.35 ± 1.74% vs. 2.12 ± 1.76%, *P* < 0.035). Conversely, the DIT after F-meal or P-meal ingestion did not correlate with BAT activity, with no difference between the two groups. Thus, BAT has a significant role in DIT after ingestion of a carbohydrate-rich meal, but hardly after ingestion either protein- or fat-rich meal.

## Introduction

Brown adipose tissue (BAT) is the major site of sympathetically activated non-shivering thermogenesis (NST) during cold exposure (cold-induced thermogenesis [CIT]) in small rodents (1). Through fluorodeoxyglucose (FDG)–positron emission tomography (PET) and computed tomography (CT), metabolically active BAT was rediscovered in adult humans (2–5). It is now confirmed that human BAT is activated by cold exposure and contributes to the regulation of whole-body energy expenditure (EE) and body fatness (6, 7). NST seen after meal ingestion, referred to as the “specific dynamic action of food,” “thermic effects of food,” “postprandial thermogenesis,” or “diet-induced thermogenesis (DIT),” is also a significant component of the total EE in our daily life. DIT refers to EE largely for digestion, absorption and metabolism/storage of ingested nutrients, and probably also for some additional responses, but its mechanisms remain poorly understood.

In 1980s, Glick et al. (8) suggested that BAT can be activated after a single meal because BAT’s respiration rate increased in 2 h after food intake in rats. We (9) found meal-induced metabolic activation of BAT in rats: that is, 30 min after meal ingestion, glucose utilization and fatty acid synthesis in BAT were increased in a sympathetically dependent manner. The role of BAT in DIT was also suggested through simultaneous 24-h recording of food intake and oxygen consumption in mice deficient of uncoupling protein 1 (UCP1), which is a key protein for BAT thermogenesis. UCP1-deficient mice showed lower whole-body oxygen consumption than wild-type mice, particularly during the eating period (10). Consistent with these studies in small rodents, we (11, 12) demonstrated in healthy humans that DIT is approximately 50% higher in participants with metabolically active BAT than in those without it, suggesting BAT’s contribution to DIT in humans (13). Human BAT activation after meal intake is directly confirmed by PET/CT using [^15^O]O_2_, [^15^O]H_2_O, and ^18^F]fluoro-thiaheptadecanoic acid radiotracers (14).

DIT is roughly 10% of the energy content of ingested meals, but it varies depending on the macronutrient composition of meal, being approximately 3% for fat, 7–8% for carbohydrate, and 25–30% for protein (15, 16). In rats, BAT is activated after ingestion of a high-carbohydrate meal, but to a lesser extent after ingestion of a high-protein or high-fat meal (17, 18). These results collectively suggest that BAT-associated DIT is largely influenced by the macronutrient composition of meal. In this study, we measured DIT after the ingestion of a carbohydrate-, protein-, or fat-rich meal in healthy human volunteers and analyzed its association with BAT activity.

## Materials and methods

### Participants

Through poster advertisements and oral communication, 41 healthy males aged 20–29 years were recruited. Before the study began, all participants provided written informed consent. They underwent FDG-PET/CT after acute cold exposure to assess BAT activity and indirect calorimetry in winter (December to March). The study protocol was approved by the institutional review board of Tenshi College.

### Test meals

The composition and energy content of the test meals were shown in Table 1. A carbohydrate-rich meal (C-meal) was prepared using commercially available nutritionally balanced foods (Calorie Mate Block, Calorie Mate Liquid, Otsuka Pharmaceutical Co., Tokyo, Japan). A protein-rich meal (P-meal) was prepared by combining the nutritionally balanced foods and a liquid containing whey protein powder (DNS Inc., Tokyo, Japan). A fat-rich meal (F-meal) was prepared by combining the nutritionally balanced foods, pork meat sausage, and a liquid containing rapeseed oil, skim milk, and soybean lecithin. These three meals yielded a carbohydrate/protein/fat (CPF) energy ratio of 51:11:38, 21:64:15, and 22:11:67, respectively. The participants consumed one of the test meals at a total energy of 430–580 kcal (7.9 kcal/kg body weight), with 100–200 mL of water as needed in 10 min.

**TABLE 1:** Nutrient composition of the test meals.

	C-meal	P-meal	F-meal
Carbohydrate (g)	63.1	26.5	28.6
Protein (g)	16.5	79.2	13.9
Fat (g)	21.2	8.1	37.2
SFA (g)	8.1	3.1	11.4
MUFA (g)	7.2	2.8	13.8
PUFA (g)	5.5	2.0	7.7
Dietary fiber (g)	4.0	1.8	1.1
Minerals (g)	1.9	2.5	1.6
Total energy (kcal)	500	493	499
CPF energy ratio	51/11/38	21/64/15	22/11/67

CPF, Carbohydrate/Protein/Fat; SFA, Saturated fatty acids; MUFA, monounsaturated fatty acids; PUFA, Polyunsaturated fatty acids.

### Fluorodeoxyglucose-positron emission tomography/computed tomography

BAT activity was measured using FDG-PET/CT, using a previously reported method (2) after slight modifications. Briefly, after fasting for 10-12 h, participants wore light clothes (T-shirt and shorts) and remained in a room at 19^°^C for 2 h. After 1 h, the participants received [^18^F]FDG (1.7 MBq/kg body weight) intravenously and remained in the same cold conditions for another hour. One hour after [^18^F]FDG administration, PET/CT was performed at 24^°^C room temperature using a dedicated PET/CT system [Aquiduo (Toshiba Medical Systems, Otawara, Japan), Biograph 16 (Siemens Medical Solutions, Knoxville, TN, USA), or Discovery PET/CT 600 (GE Healthcare, Waukesha, WI, USA)]. Detectable [^18^F]FDG uptake into the supraclavicular BAT was assessed by visual judgment. In parallel, the [^18^F]FDG uptake was quantified as the maximal standardized uptake value (SUV_*max*_).

### Indirect calorimetry

Postprandial thermogenesis was estimated by measuring whole-body EE before and after meal ingestion with the use of a respiratory gas analyzer connected to a ventilated hood (AR-1, Arco System, Kashiwa, Chiba, Japan). Briefly, after fasting for 10–14 h, the participants relaxed on a bed while wearing light clothing in a room at 27^°^C, and oxygen consumption and carbon dioxide production were continuously recorded for 30 min. In the last 10-min period, the stable value was used to calculate the basal EE. EE was calculated form oxygen consumption and carbon dioxide production by the equation of Weir (19). Protein oxidation was assumed to be 21.4% of resting EE. Then, the participants had one of the test meals (C-meal, P-meal, or F-meal) with a total energy of 7.9 kcal/kg body weight in 10 min. After 15, 45, 75, and 105 min, the respiratory gas parameters were recorded for 20 min, and the EE during the last 10 min was calculated.

### Statistical analysis

Values are presented as the mean ± standard deviation. All statistical data were analyzed using SPSS (version 26, IBM Japan, Tokyo, Japan). The EE values were examined using two-way analysis of variance (ANOVA) for repeated measures based on a within-participant factor (time) and a between-participant factor (BAT). The differences in DIT after the C-, P-, and F-meals were compared by ANOVA followed by Tukey’s *post-hoc* test. The differences between the High- and Low-BAT groups were compared by Student’s *t*-test. *P*-values of less than 0.05 were considered significant.

## Results

### Participant characteristics

**FIGURE 1:**
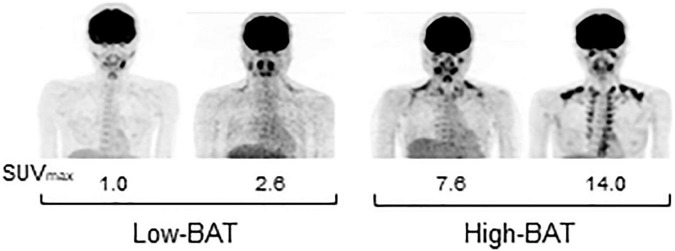
FDG-PET images of participants in the Low- and High-BAT groups. PET/CT was performed after 2-h cold exposure, and [^18^F]FDG uptake into the supraclavicular BAT was quantified as the maximal standardized uptake value (SUV_*max*_).

A total of 41 young male participants underwent FDG-PET/CT, anthropometrics, and body composition analysis and then participated in indirect calorimetry before and after test meal ingestion (Table 2). Eleven out of those who consumed the C-meal also participated in the calorimetry for the P-meal. The participants were divided into two groups: those with lower (Low-BAT group) and higher (High-BAT group) than the median value of SUV_*max*_ (Figure 1). The mean SUV_*max*_ in the High-BAT group was 5.8–10 times higher than that in the Low-BAT group (Table 2). Age, anthropometric parameters, and resting EE before meal ingestion were not significantly different between the two groups.

**TABLE 2:** Participant characteristics.

C-meal			

	All	Low BAT	High BAT

*N*	19	9	10
Age (yo)	22.6 ± 3.0	21.4 ± 0,9	23.7 ± 3.8
Height (cm)	171.9 ± 7.3	173.3 ± 5.3	170.6 ± 8.7
Body weight (kg)	64.0 ± 9.1	65.0 ± 5.7	63.1 ± 11.7
BMI (kg/m^2^)	21.6 ± 2.1	21.7 ± 1.7	21.5 ± 2.6
Body fat (%)	16.2 ± 4.2	16.3 ± 4.1	16.1 ± 4.6
Fat free mass (kg)	53.4 ± 6.0	54.4 ± 4.7	52.5 ± 7.1
SUV_*max*_	7.9 ± 10.0	1.9 ± 1.1	13.3 ± 11.4**
EE (kcal/day)	1,549 ± 193	1,586 ± 195	1,515 ± 195
EE (kcal/kgBW/day)	24.4 ± 2.6	24.6 ± 3.0	24.2 ± 2.3

**P-meal**			

	**All**	**Low BAT**	**High BAT**

*N*	15	7	8
Age (yo)	22.7 ± 3.6	21.7 ± 1.0	23.6 ± 4.6
Height (cm)	171.8 ± 7.0	174.4 ± 6.8	169.4 ± 7.2
Body weight (kg)	63.3 ± 8.7	64.0 ± 5.9	62.7 ± 7.9
BMI (kg/m^2^)	21.5 ± 12.7	21.1 ± 1.7	21.8 ± 2.4
Body fat (%)	15.6 ± 4.3	16.2 ± 3.1	15.0 ± 4.3
Fat free mass (kg)	53.2 ± 5.7	53.7 ± 5.2	52.8 ± 5.0
SUV_*max*_	5.7 ± 5.4	1.6 ± 0.9	9.3 ± 5.1**
EE (kcal/day)	1,422 ± 173	1,451 ± 181	1,395 ± 161
EE (kcal/kgBW/day)	22.6 ± 2.4	22.7 ± 2.6	22.5 ± 2.2
**F-meal**			

	**All**	**Low BAT**	**High BAT**

*N*	18	9	9
Age (yo)	23.6 ± 1.2	24.0 ± 1/3	23.0 ± 0.9
Height (cm)	173.2 ± 4.8	172.6 ± 4.3	173.7 ± 5.4
Body weight (kg)	62.9 ± 8.9	64.8 ± 9.1	61.1 ± 8.7
BMI (kg/m^2^)	21.0 ± 2.7	21.7 ± 3.1	20.2 ± 2.2
Body fat (%)	16.9 ± 5.8	18.0 ± 6.5	15.8 ± 5.0
Fat free mass (kg)	51.4 ± 4.7	52.6 ± 3.1	51.2 ± 6.0
SUV_*max*_	8.3 ± 9.7	1.5 ± 0.9	15.0 ± 9.8**
EE (kcal/day)	1,506 ± 156	1,540 ± 154	1,473 ± 159
EE (kcal/kgBW/day)	24.1 ± 1.8	23.9 ± 1.8	24.3 ± 1.9

Participants were divided into two groups: those with lower (Low-BAT group) and higher (High-BAT group) than the median value of SUVmax (4.1 for C-meal, 4.1 for P-meal, and 3.3 for F-meal).

Values are expressed as mean ± SD.

**P < 0.01 vs. Low-BAT.

### Diet-induced thermogenesis based on the test meals

**FIGURE 2:**
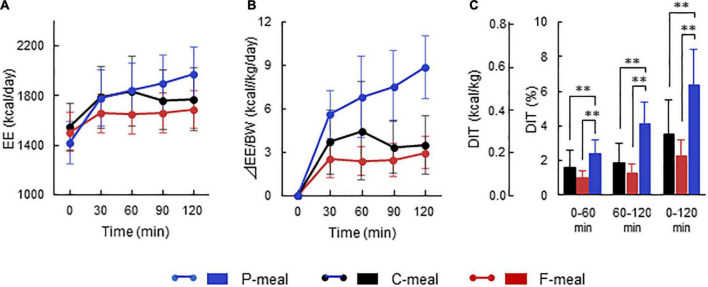
Energy expenditure and diet-induced thermogenesis after ingestion of test meals. Whole-body energy expenditure (EE) was measured after ingestion of a carbohydrate-rich C-meal **(Black)**, a protein-rich P-meal **(Blue)**, and a fat-rich F-meal **(Red)** at 7.9 kcal/kg body weight (BW). **(A)** Whole-body EE. **(B)** Postprandial change in EE calculated as relative to BW. **(C)** Diet-induced thermogenesis (DIT) calculated as the area under the curve in **(B)** and expressed as % of ingested energy. Values are expressed as mean ± *SD*. ^**^P < 0.01.

Figure 2 shows whole-body EE before and after meal ingestion in all participants regardless of having low- and high-BAT activities. After meal ingestion, EE increased rapidly at 30 min and thereafter (Figure 2A). Given that the absolute values of basal EE and postprandial increase (calculated as the difference from the basal EE) vary depending on the body size and ingested amount of energy, the ingested meal amount was adjusted to 7.9 kcal/kg body weight in the present study. Thus, the postprandial increase calculated in relation to body weight was higher after the P-meal than after the C-meal and F-meal (Figure 2B), and ANOVA revealed highly significant effects of time, meal, and time × meal interaction (*P* < 0.001). This result was also confirmed when postprandial increase was integrated as the area under the curve and expressed as% of ingested energy. DIT thus calculated at 0–60 and 60–120 min was highest after the P-meal and lowest after the F-meal, and that at 0–120 min was 6.44 ± 2.01%, 3.49 ± 2.00%, and 2.32 ± 0.90% after the P-, C-, and F-meals, respectively (Figure 2C).

### Effects of brown adipose tissue activity on diet-induced thermogenesis

**FIGURE 3:**
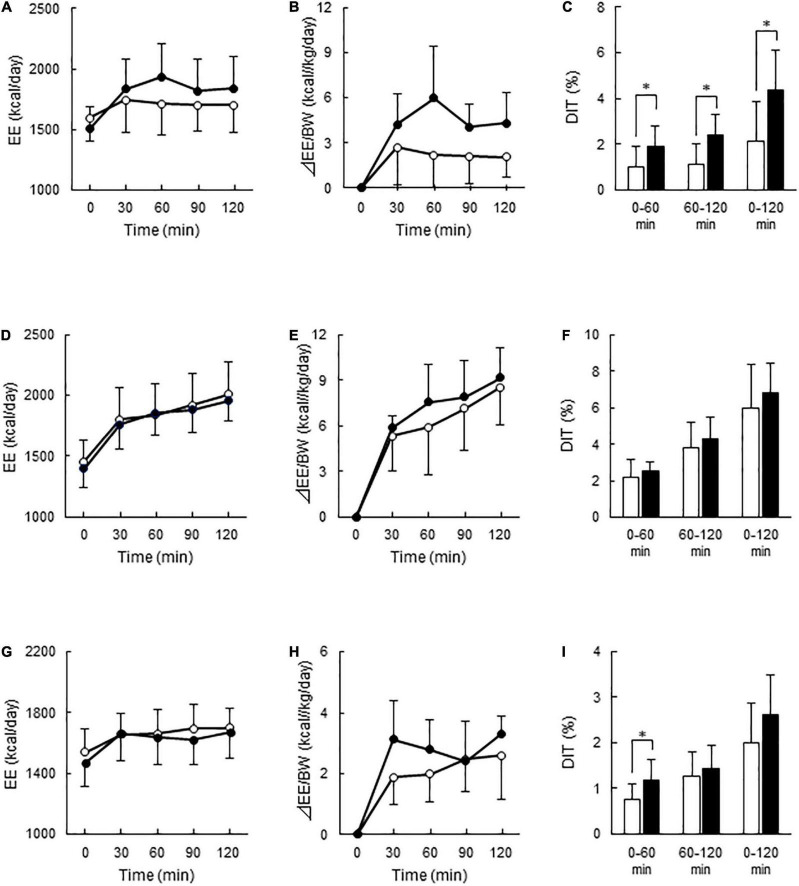
Energy expenditure and diet-induced thermogenesis after meal ingestion in the Low- and High-BAT groups. Whole-body energy expenditure (EE) was measured after ingestion of a carbohydrate-rich C-meal **(A–C)**, a protein-rich P-meal **(D–F)**, and a fat-rich F-meal **(G–I)** at 7.9 kcal/kg body weight (BW). Open circles and columns: Low-BAT group. Filled circles and columns: High-BAT group. **(A,D,G)** Whole-body EE. **(B,E,H)** Postprandial change in EE calculated as relative to BW. **(C,F,I)** Diet-induced thermogenesis (DIT) calculated as the area under the curve in **(B,E,H)** and expressed as % of ingested energy. Values are expressed as mean ± *SD*. *P < 0.05.

**FIGURE 4:**
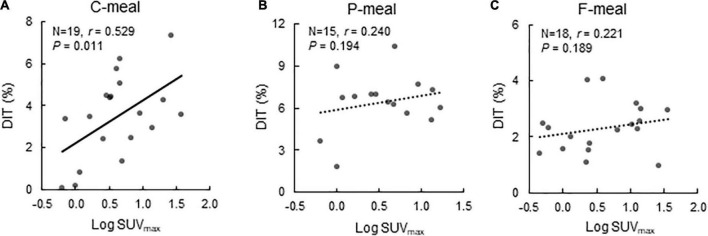
Correlation between BAT activity and diet-induced thermogenesis. Correlation between BAT activity expressed as Log SUV_*max*_ and diet-induced thermogenesis (DIT) after ingestion of a carbohydrate-rich C-meal **(A)**, a protein-rich P-meal **(B)**, and a fat-rich F-meal **(C)**. r: Correlation coefficient.

After C-meal ingestion, EE seemed to increase with time more in the High-BAT group than in the Low-BAT group (Figure 3A), and ANOVA revealed a significant time × BAT interaction in postprandial EE increase (*P* = 0.038, Figure 3B). The integrated DIT at 0–120 min was significantly greater in the High-BAT group than in the Low-BAT group (4.35 ± 1.74% vs. 2.12 ± 1.76%, *P* < 0.05) (Figure 3C). Moreover, DIT showed a significant positive correlation with BAT activity expressed as Log SUVmax (*P* = 0.011, Figure 4A). A significant positive correlation was also found between BAT activity and DIT at 0–60 min (*P* = 0.013) and 60–120 min (*P* = 0.010).

After P-meal ingestion, EE, postprandial increase in EE, and DIT were almost comparable between the two groups (Figures 3D–F), and DIT did not significantly correlate with BAT activity (*P* = 0.194, Figure 4B). After F-meal ingestion, EE seemed to increase with time similarly in the two groups (Figure 3G), but ANOVA revealed a significant time × BAT interaction (*P* = 0.036) in postprandial increase (Figure 3H). DIT at 0–60 min was significantly higher (*P* < 0.05) in the High-BAT group than in the Low-BAT group (Figure 3I). However, DIT at 60–120 and 0–120 min showed no significant difference between such groups (Figure 3I). In addition, DIT showed no significant correlation with BAT activity (*P* = 0.189, Figure 4C), although DIT at 0–60 min tended to correlate positively with BAT activity (*P* = 0.064).

## Discussion

DIT is roughly 10% of the energy content of ingested meal, and it varies depending on meal composition, being 3% for fat, 7–8% for carbohydrate, and 25–30% for protein (15, 16). Similarly, our results demonstrated that DIT was highest after the P-meal (6.4%) and lowest after the F-meal (2.3%), and approximately 3.5% after the C-meal. Our DIT values are lower than the reported values because of the duration of the measurement. The above-mentioned DIT was obtained for 6–10 h after meal ingestion, whereas the DIT in our study was measured for 2 h after meal ingestion. We (11, 12) previously reported that DIT with a mixed meal containing 60% carbohydrate was approximately 8.6% when measured for 5 h using a whole-room human calorimeter. In the present study, EE was measured for 2 h using a respiratory gas analyzer connected to a ventilated food; thus, collecting a stable value for a longer time period was difficult, probably because of the increasing stress of repeated immobilization for the measurement.

The present study also demonstrated that DIT after the C-meal was almost twice higher in the High-BAT group than in the Low-BAT group. Moreover, DIT showed a highly significant correlation with BAT activity. These results are consistent with our previous report that DIT with a mixed meal containing 60% carbohydrate was approximately 1.5 times higher in participants with active BAT than in those without it (11, 12), suggesting that a substantial component of DIT after a C-meal is attributable to BAT activation. Vosselman et al. (20) measured BAT activity using FDG-PET/CT in healthy volunteers 120 min after intake of a carbohydrate-rich meal (CPF energy ratio of 78:12:10), and found that postprandial FDG uptake into BAT was much lower than cold-induced uptake, whereas whole-body EE was comparable. Vrieze et al. (21) also reported an unexpected reduction in FDG uptake into BAT compared with that after overnight fasting. Although these results seem to be in conflict with the idea of postprandial activation of BAT thermogenesis, they can be explained by increased insulin-stimulated FDG uptake into skeletal muscle, which reduces FDG bioavailability for BAT, which in turn leads to underestimation of BAT activity. Actually, using ^15^O[O_2_, H_2_O]-PET instead of FDG-PET, Din et al. (14) demonstrated the activation of BAT thermogenesis 15 min after ingestion of a meal containing 58% carbohydrate.

Conflicting with our results, Loeliger et al. (22) reported that human BAT activity assessed by FDG-PET/MRI was associated with EE increase after mild cold exposure, but not with that after an oral glucose load; thus, they argued that DIT is independent from BAT activation. Such apparent discrepancy still cannot be explained clearly, but it may have resulted from the difference in the loaded substances and their energy content. We gave a nutritionally balanced food with a CPF energy ratio of 51:11:38 at a dose of 7.9 kcal/kg body weight (total energy of 430–580 kcal), whereas they gave 75 g (300 kcal) of glucose dissolved in tap water solution. They also did not show the participants’ body weight; thus, the energy dose could not be calculated. Another possible reason is that the rate for the digestion and absorption of our C-meal is different from that of their glucose solution, thereby affecting the results. In fact, postprandial EE increased for at least 2 h in our study, whereas that in their study increased for only 1 h.

Contrary to C-meal ingestion, P-meal or F-meal ingestion showed that DIT was not significantly different between the Low- and High-BAT groups. Consistently, the DIT in P-meal or F-meal showed no correlation with BAT activity. The DIT 1 h after the F-meal was slightly but significantly higher in the High-BAT group than in the Low-BAT group, suggesting that BAT may contribute to fat-induced DIT only during the initial phase. These results seem compatible with those reported in rats in which the *in vitro* respiration rate of BAT was lower after P- or F-meals than after C-meals (17, 18). Thus, thermogenesis by BAT after protein or fat ingestion is considerably less, or negligible, compared with that after carbohydrate ingestion.

BAT thermogenesis is activated through the sympathetic nerve (SN) and β-adrenoceptor (βAR) axis. Based on this well-accepted view, this axis may also play a key role in DIT. In both experimental animals and humans, the SN activity assessed from the plasma levels of noradrenaline (NA) and tissue NA turnover decreases during fasting but increases immediately after food intake (23–26). More notably, postprandial SN activation is higher after carbohydrate ingestion than after protein or fat ingestion (26–29). These results are quite consistent with our present results and support the idea that the SN–βAR–BAT axis is activated after C-meal ingestion but only slightly after P- or F-meal ingestion, thereby contributing substantially to DIT. However, reports on the effect of βAR blockers on DIT in humans are conflicting; some demonstrated decreased DIT, while others showed no effects (30–32). Such discrepancy still could not be explained clearly, but it may be related to individual differences in BAT activity, that is, blockade of βAR may suppress DIT only in participants with high-BAT activity. All previous studies did not take the participants’ BAT activity into consideration and showed the combined data of all participants.

In addition, some mechanisms different from and/or in combination with the SN–βAR axis are likely involved in human DIT. One of the possible factors is secretin. Li et al. (33) found that the secretin receptor in murine brown adipocytes was highly expressed and that secretin activated BAT thermogenesis *in vitro* and *in vivo*. They also confirmed that the increment of plasma secretin levels induced by a single meal positively correlated with oxygen consumption and fatty acid uptake rates in human BAT. Therefore, meal-associated increase in circulating secretin activates BAT thermogenesis by binding to the receptor in brown adipocytes. Direct evidence for the thermogenic action of secretin on human BAT was obtained using FDG-PET/CT after secretin infusion, which significantly increased [^18^F]FDG uptake in supraclavicular BAT. However, to our knowledge, the insight into whether or not secretin action blockade suppresses DIT in humans remains unreported. Physiologically, acids in the duodenal luminal chime stimulate secretin secretion, but the mechanism on how the macronutrient composition of ingested meal affects the duodenal acidity and/or plasma secretin levels is still undetermined. Further human studies are needed to clarify the mechanisms of BAT-associated DIT, with references to the roles of secretin, other gastrointestinal factors, and the SN–βAR axis.

There are some limitations to our study. First, the study was to use a parallel design, not a crossover design which would be better to compare the effect of different meals. In fact, 11 out of 19 participants who consumed the C-meal also participated in the calorimetry for the P-meal, but none of them could participate in that for the F-meal, mainly because of different study years. However, this would not be critical in our study, the main objective of which was to examine the effect of BAT on DIT after individual meals. Another limitation to the present study is that DIT was assessed from the EE measurement only for 2 h after meal ingestion. As DIT is known to persist for 5 h or more, we may have missed any differences that occur at the later phase.

## Conclusion

In conclusion, DIT after a single C-meal in healthy human participants was higher in those with higher BAT activity than in those with lower BAT activity. The DIT after a P-meal and a F-meal was higher and lower, respectively, than that after a C-meal; however, it had no correlation with BAT activity. These results suggest that BAT has a significant role in DIT after carbohydrate ingestion but hardly after protein or fat ingestion. Thus, dietary carbohydrate may be the most effective macronutrient for postprandial activation of BAT. To obtain further evidence for this, direct measurement of EE by BAT itself is needed, for example, by using ^15^O[O_2_, H_2_O]-PET.

## Data availability statement

The raw data supporting the conclusions of this article will be made available by the authors, without undue reservation.

## Ethics statement

The studies involving human participants were reviewed and approved by the Institutional Review Board of Tenshi College. The patients/participants provided their written informed consent to participate in this study.

## Author contributions

SA, MM, and MS conceived and designed the study. SA, MM, TY, TH, TK, and IO performed the experiments. SA, MM, TY, and MS performed data analysis and interpretation. MS wrote the manuscript. All authors read and approved the manuscript.
